# When Childhood Control Slips Away: How Parental Affection and Abuse Shape Adult Anxiety and Depression

**DOI:** 10.1002/cpp.70232

**Published:** 2026-02-03

**Authors:** Chui Pin Soh, Kristin L. Szuhany, Nur Hani Zainal

**Affiliations:** ^1^ Department of Psychology National University of Singapore (NUS) Singapore; ^2^ Department of Psychiatry New York University (NYU) Grossman School of Medicine New York City New York USA

**Keywords:** anxiety, childhood abuse, depression, mediation analysis, parental affection, sense of control, structural equation modelling

## Abstract

Childhood parental affection and abuse may shape vulnerability to generalized anxiety disorder (GAD) and major depressive disorder (MDD) in adulthood through personal mastery and perceived constraints. This three‐wave 18‐year longitudinal study tested whether sense‐of‐control dimensions mediated the effects of early parental experiences on later GAD and MDD symptoms (*N* = 3294; 54.9% women; mean age = 45.6 years, *SD* = 11.4, range = 20–74 years; 89.7% White compared to African American, Asian/Pacific Islander, Native American and other). Structural equation models showed that lower parental affection and higher abuse at Time 1 predicted greater perceived constraints at Time 2 (Cohen's *d* = −0.396 to 0.510), which in turn predicted greater GAD and MDD severity at Time 3 (*d* = 0.463 to 0.754). Perceived constraints significantly mediated the links between childhood parental experiences and adult symptom severity for both GAD (*d* = −0.269–0.319; percentage mediated: 30.0%–69.2%) and MDD (*d* = −0.343–0.422; 11.0%–44.9%), whereas mastery did not. Mediated effects were somewhat stronger for maternal (11.4%–69.2%) than paternal (11.0%–51.5%) experiences. These findings underscore perceived constraints as a critical mechanism linking childhood parental experiences to later anxiety and depression. Interventions that address maladaptive beliefs about sense of control may improve long‐term outcomes for adults exposed to early adversity.

## Summary

- Perceived constraints are a central psychological mechanism linking early parenting experiences to adult anxiety and depression
- Beliefs about external constraints emerged as a more salient mechanism in relation to anxiety and depressive symptoms than self‐efficacy beliefs.
- Psychotherapeutic intervention may benefit from assessing and addressing control‐related beliefs.
- Mechanism‐informed formulation may help guide psychotherapy for clients with childhood parental maltreatment.

## 1 Introduction

Anxiety and depressive disorders are major global public health concerns (COVID‐19 Mental Disorders Collaborators 2021). Worldwide, lifetime prevalence is about 3.7% for generalized anxiety disorder (GAD) and 4.7% for major depressive disorder (MDD; GBD 2019 Mental Disorders Collaborators. 2022; Ruscio et al. 2017). GAD and MDD frequently co‐occur, show shared genetic liability in cross‐sectional work (Hettema 2008; Kendler et al. 2007) and confer bidirectional risk in longitudinal meta‐analytic evidence (Jacobson et al. 2017). Both disorders impair functioning at work, in interpersonal domains and in executive function (EF; el‐Guebaly et al. 2007; Hames et al. 2013; Rapaport et al. 2005; Shin and Newman 2019; Zainal and Newman 2021). Identifying protective and risk factors for GAD and MDD may therefore aid early prevention and treatment.

The development of GAD and MDD often traces back to early childhood experiences. Positive experiences, such as supportive parental relationships, serve as protective factors through biopsychosocial pathways (Li et al. 2016; Moffitt et al. 2007; Yap and Jorm 2015). Affectionate and supportive parents foster emotional, social and behavioural growth by nurturing exploration, autonomy and challenge‐seeking (Soenens and Vansteenkiste 2020; Watkins et al. 2007), thereby strengthening self‐esteem and perceived control that promote prosocial behaviour, problem‐solving and goal‐setting (Lachman and Firth 2004; Ryan et al. 2016). Consistent with these mechanisms, parental affection and adaptive parent–child relations predicted better mental well‐being and psychological functioning (Chen et al. 2019; Stafford et al. 2016). Meta‐analytic evidence showed small to moderate cross‐sectional and prospective associations between parental warmth, particularly autonomy granting, and lower internalizing symptoms (Pinquart 2017; Yap and Jorm 2015). Longitudinal studies similarly revealed inverse links between parental affection and depression and anxiety severity (Bartek et al. 2021; Chen and Harris 2019; Wang et al. 2021).

Conversely, child abuse (e.g., maltreatment and family violence) is a key risk factor for elevated adulthood GAD and MDD symptoms (Mandelli et al. 2015; Shafiei et al. 2022). Trauma‐related dysregulation of the stress response can heighten cortisol reactivity over time, compromising stress‐sensitive brain regions supporting executive function (EF) and emotion regulation (ER; Hosseini‐Kamkar et al. 2021). Consistent with this model, parental abuse and neglect are linked to poor self‐esteem and self‐acceptance (Finzi‐Dottan and Karu 2006; Sanghvi et al. 2023), substance dependence (Wu et al. 2010), greater dispositional anger expression (Win et al. 2021) and impaired ER (Marr et al. 2022). Meta‐analyses indicated that exposure to childhood sexual, emotional or physical abuse conferred two to three times higher odds of adult suicide attempts (Angelakis et al. 2019) and small to medium associations with adulthood anxiety, depression and suicidal ideation (Xiao et al. 2023). These findings underscore the need to delineate how early parental affection and abuse relate to later GAD and MDD and to identify mechanisms that can guide targeted prevention and treatment.

Perceived control, central to adaptive functioning, plausibly links early experiences to later psychopathology. It comprises two related constructs: *mastery* or self‐efficacy, the belief in one's capacity to enact goal‐directed behaviours, and *constraints*, the belief that external forces govern life circumstances (Bandura and Ramachaudran 1994; Infurna and Mayer 2015; Lachman and Weaver 1998; Skinner 1996). Supportive parenting fosters mastery and the expectation that one can influence outcomes, which may protect against later anxiety and depression (Lachman and Firth 2004; Lachman and Weaver 1998). Conversely, parental maltreatment and neglect engender helplessness and uncontrollability, disrupt cognition and brain function and align with the triple vulnerability account that adverse childhood experiences reduce perceived agency (Barlow et al. 2014; Lachman 2006; Lund et al. 2020; Pilkington et al. 2021). Although such beliefs may be adaptive within abusive contexts, they predict poorer psychological outcomes in adulthood when the threat is absent (Hoare et al. 2016; Jacobson et al. 2001; Riggs 2019). Together, the dual impact of low parental affection and high abuse on perceived control underscores its role in shaping long‐term GAD and MDD severity.

Although control beliefs are widely linked to MDD and GAD, relatively few studies test their mediating role between child abuse and subsequent mental health. Cross‐sectional work showed that mastery mediates associations between childhood abuse and health problems in older adults (Sachs‐Ericsson et al. 2011) and that perceived control mediated links between childhood physical violence and adult depressive symptoms (Shaw and Krause 2002); retrospective family life happiness relates to mastery, which in turn was related to late‐life cognitive function (Lee and Schafer 2021). However, cross‐sectional mediation is biased and cannot establish causality (Maxwell and Cole 2007). Prospective evidence indicated that greater childhood abuse predicts lower perceived control days later in college students (Nguyen‐Feng et al. 2017) and, over years, decreased mastery and increased constraints in mid‐to‐late adulthood, which predicted greater distress and mortality, respectively (Elliot et al. 2018). In children, perceived external control mediated the link between neglect and later internalizing problems across four years (Bolger and Patterson 2001).

Previous studies had several limitations that the present study aimed to address. Most lacked the recommended minimum of three time points for robust mediation analysis (Maxwell and Cole 2007). Parental affection and abuse were often combined into a single construct (Elliot et al. 2018; Nguyen‐Feng et al. 2017; Sachs‐Ericsson et al. 2011), obscuring distinct maternal and paternal influences on the development of GAD and MDD (Bornstein 2015; Cox and Paley 1997). Likewise, perceived control was frequently treated as a single dimension, despite evidence that mastery and constraints differentially affect mental health (Infurna and Mayer 2015). Accordingly, this study separately examined maternal and paternal affection and abuse, as well as the distinct roles of mastery and constraints. Clarifying these specific effects may inform targeted prevention and treatment for individuals with or at risk for GAD and MDD.

Person‐centred care prioritizes clients' lived experiences to tailor treatments that support individuals and address systemic influences on well‐being (Pinho et al. 2021). Past research showed that maternal and paternal involvement differentially predicted adolescent mood problems (Manuele et al. 2023). Mothers' greater emotional sensitivity in daily caregiving may better detect subtle distress or withdrawal (Pinquart 2017). Paternal play‐based engagement fosters self‐regulation, which can reduce internalizing and externalizing behaviours (Dubeau et al. 2013; Jones et al. 2000).

We tested whether trait‐level mastery and constraints mediated the 18‐year effects of parental affection and abuse on adulthood GAD and MDD severity. We hypothesized that higher parental affection and lower abuse would increase mastery and reduce constraints, which in turn would predict lower GAD and MDD severity. We further posited differential mediating effects of low mastery and high constraints depending on whether childhood experiences were maternal or paternal in origin.

## 2 Method

### 2.1 Study Design and Participants

We analysed three MIDUS assessment waves spaced about 9 years apart: 1995–1996 (T1), 2004–2005 (T2) and 2012–2013 (T3; Brim et al. 2017; Ryff et al. 2019; Ryff et al. 2021). No additional ethics approval was required because MIDUS is publicly available (http://tinyurl.com/icpsrmidus). The original study had ethics approval from four universities, and all participants provided informed consent. Original sample sizes were 7108 (T1), 4963 (T2) and 3294 (T3); analyses focused on the 3294 individuals with complete data across all waves. At T1, participants were 20 to 74 years old, 54.6% female and 46.8% college educated; 89.01% identified as White, with the remainder African American, Asian, Native American or other. Sociodemographic details appear in Table S1. This study was not preregistered.

### 2.2 Procedures

We analysed participants with telephone or self‐report assessments of GAD and MDD severity at T1 and T3; parental affection and abuse at T1; and mastery and constraints at T2 (procedures: https://midus.wisc.edu/).

### 2.3 Measures

#### 2.3.1 Retrospectively Recalled Childhood Parental Affection at T1

Parental affection during childhood was retrospectively measured with the Parental Support Scale (Rossi 2001), comprising separate 6‐item maternal and paternal subscales rated from 1 (*Not at all*) to 4 (*A lot*). Example items included ‘how much love and affection did your mother/father give you?’ and ‘how much effort did your mother/father put into watching over you and ensuring a good upbringing?’ Internal consistency was high for maternal (*α* = 0.91) and paternal affection (*α* = 0.93), with higher scores indicating stronger affection. The scale showed good convergent and discriminant validity, correlating positively with well‐being and minimally with unrelated constructs (Chen et al. 2019; Moran et al. 2018).

#### 2.3.2 Retrospectively Recalled Childhood Abuse at T1

Parental child abuse was retrospectively assessed with the Revised Conflict Tactics Scale (CTS2; Straus et al. 1996), which measured the frequency of childhood emotional, physical and severe physical abuse on a 4‐point Likert scale (1, *Never* to 4, *Often*). Emotional items include ‘threatened to hit you; smashed or kicked something in anger; did or said something to spite you; insulted or swore at you; stomped out of the room; sulked or refused to talk to you’. Physical items included ‘pushed, grabbed, or shoved you; slapped you; threw something at you’. Severe physical items included ‘kicked, bit, or hit you with a fist; hit or tried to hit you with something; beat you up; choked you; burned or scalded you’. Higher scores indicated greater abuse. Internal consistency was acceptable for emotional (*α* = 0.73), physical (*α* = 0.71) and severe physical (*α* = 0.75) domains. The CTS2 had strong psychometrics across populations and cultures (Chapman and Gillespie 2019; Jones et al. 2017), showed convergent validity via associations between physical assault severity and conviction rates (Loinaz 2013) and correlated strongly with the Abusive Behaviours Checklist, supporting criterion validity (Ronan et al. 2013).

#### 2.3.3 Personal Mastery and Perceived Constraints at T2

The Sense of Control scale (Lachman and Weaver 1998) assessed two control‐belief dimensions. Personal mastery (*α* = 0.73), indexed by four items (e.g., ‘When I really want to do something, I usually find a way to succeed at it’), reflected self‐efficacy in achieving goals. Perceived constraints (*α* = 0.85), indexed by eight items (e.g., ‘I have little control over the things that happen to me’), reflected beliefs that external factors govern outcomes. Items are rated on a 7‐point Likert scale; higher scores indicate stronger mastery or constraints.

#### 2.3.4 GAD and MDD Symptom Severity at T1 and T3

The Composite International Diagnostic Interview–Short Form (CIDI‐SF; Kessler et al. 1998) assessed GAD and MDD severity based on *DSM‐III‐R* criteria (American Psychiatric Association 1987). GAD severity ranged from 0 to 10, reflecting symptoms such as irritability, restlessness, sleep disturbance, fatigue, muscle tension, inattention and forgetfulness, coded 1 (‘worries most days’) or 0 (*Never*). MDD severity ranged from 0 to 7, indexing concentration problems, low self‐worth, sleep or appetite changes, fatigue, anhedonia and thoughts of death, coded 1 (*Yes*) or 0 (*No*). The CIDI‐SF demonstrated high sensitivity (GAD = 96.6%, MDD = 89.6%) and specificity (GAD = 99.8%, MDD = 93.9%) (Kessler et al. 1998).

### 2.4 Data Analysis

Incomplete data comprised 3.5% and were handled with multiple imputation, the benchmark method when missingness is presumed random (Lee and Shi 2021). Longitudinal SEM was conducted in RStudio using *lavaan* (R 4.3.2; Rosseel 2012). Model fit was evaluated with CFI (Bentler 1990), *χ* ^2^ with *df* and *p*‐value (Hu and Bentler 1999), RMSEA (Steiger 1990) and SRMR (Byrne 2001; Hu and Bentler 1999). Values of CFI ≥ 0.90 and RMSEA and SRMR ≤ 0.10 indicated acceptable fit, whereas values of CFI ≥ 0.95 and RMSEA and SRMR ≤ 0.080 reflected excellent fit.

We tested indirect effects using the product‐of‐coefficients approach (*a × b*): T1 parental affection or abuse predicting T2 mastery or constraints (*a* path), and T2 mastery or constraints predicting T3 GAD or MDD severity (*b* path). We used 2000‐sample bootstrapping (Preacher and Hayes 2008) and reported unstandardized *β*, 95% CIs, *p* values and Cohen's *d*. Mediation effect sizes were the proportion of the indirect effect (*a × b*) relative to the total effect (*c* = *a* × *b* + *c*') (Preacher and Kelley 2011; Wen and Fan 2015). Baseline GAD or MDD severity was covaried in the respective models; T1 mastery and constraints were not included because including them could block the mediation pathway (D'Onofrio et al. 2020). OSM Figure S1 depicts the SEM example in which childhood parental affection predicts T2 mastery, which in turn predicts T3 GAD severity, while adjusting for baseline GAD. Sensitivity analyses tested robustness to age, sex, education and race.

## 3 Results

### 3.1 T1 Parental Child Affection Predicting T3 GAD Severity via T2 Mastery

OSM Figure S2 summarized the bivariate associations. The mediation models showed acceptable fit for maternal affection (*χ* ^2^(429) = 1967.13, *p* < 0.001; CFI = 0.911; RMSEA = 0.055) and paternal affection (*χ* ^2^(429) = 5092.20, *p* < 0.001; CFI = 0.923; RMSEA = 0.053). As shown in Table 1 (Part A), lower maternal (*d =* 0.550, *p* < 0.001) and paternal affection (*d =* 0.578, *p* < 0.001) at T1 predicted reduced mastery at T2, which in turn predicted higher T3 GAD severity: maternal path (*d =* −0.248, *p =* 0.010); paternal path (*d =* −0.249, *p =* 0.010). Indirect effects via reduced mastery were significant for maternal (*d =* −0.227, *p =* 0.019) and paternal affection (*d =* −0.232, *p =* 0.016), accounting for 27.8% and 26.8% of the total effects, respectively.

**TABLE 1:** T1 childhood parental affection predicting T3 GAD and MDD severity via T2 personal mastery.

	Maternal affection as predictor		Paternal affection as predictor	
	*β* (95% CI)	*d*	*β* (95% CI)	*d*
A. T3 GAD severity as outcome Regression estimates				
T1 affection to T2 Mastery	0.100*** (0.065, 0.134)	0.550	0.091*** (0.061, 0.121)	0.578
T2 mastery to T3 GAD severity	−0.005* (−0.010, −0.001)	−0.248	−0.005* (−0.010, −0.001)	−0.249
T1 affection to T3 GAD severity	0.002 (−0.001, 0.006)	0.119	0.002 (−0.001, 0.006)	0.138
T1 GAD severity to T3 GAD severity	0.272*** (0.179, 0.365)	0.555	0.271*** (0.179, 0.364)	0.555
Indirect and total effects				
Indirect effect	−0.001* (−0.001, 0.000)	−0.227	0.000* (−0.001, 0.000)	−0.232
Total effect	0.002 (−0.002, 0.006)	0.094	0.002 (−0.001, 0.005)	0.111
B. T3 MDD severity as outcome				
Regression estimates				
T1 affection to T2 mastery	0.100*** (0.066, 0.134)	0.694	0.091*** (0.061, 0.121)	0.728
T2 mastery to T3 MDD severity	−0.008 (−0.015, 0.000)	−0.227	−0.008 (−0.016, 0.000)	−0.230
T1 affection to T3 MDD severity	−0.004 (−0.010, 0.002)	−0.149	−0.002 (−0.008, 0.003)	−0.102
T1 MDD severity to T3 MDD severity	0.271*** (0.219, 0.323)	1.254	0.272*** (0.221, 0.324)	1.259
Indirect and total effects				
Indirect effect	−0.001 (−0.002, 0.000)	−0.213	−0.001 (−0.001, 0.000)	−0.216
Total effect	−0.005 (−0.011, 0.002)	−0.176	−0.003 (−0.008, 0.002)	−0.134

* *p* < 0.05.

** *p* < 0.01.

*** *p* < 0.001.

Abbreviations: CI, confidence interval; *d*, effect size; GAD, generalized anxiety disorder; MDD, major depressive disorder; *p*, *p*‐value; T1, time 1; T2, time 2; T3, time 3; *β*, factor loading or regression estimate.

### 3.2 T1 Parental Child Affection Predicting T3 MDD Severity via T2 Mastery

The mediation models showed excellent fit for maternal affection (*χ* ^2^(270) = 2429.36, *p* < 0.001; CFI = 0.972; RMSEA = 0.046) and paternal affection (*χ* ^2^(270) = 2030.15, *p* < 0.001; CFI = 0.979; RMSEA = 0.041). As shown in Table 1 (Part B) and OSM Figure S2, lower maternal (*d* = 0.694, *p* < 0.001) and paternal affection (*d* = 0.728, *p* < 0.001) at T1 predicted reduced mastery at T2. However, poorer mastery did not significantly predict higher T3 MDD severity: maternal (*d* = −0.227, *p* = 0.062); paternal (*d* = −0.230, *p* = 0.059). Corresponding indirect effects were nonsignificant: maternal (*d* = −0.213, *p* = 0.081); paternal (*d* = −0.216, *p* = 0.075).

### 3.3 T1 Parental Child Affection Predicting T3 GAD Severity via T2 Constraints

The mediation models showed acceptable fit for maternal affection (*χ* ^2^(555) = 5612.01, *p* < 0.001; CFI = 0.915; RMSEA = 0.048) and paternal affection (*χ* ^2^(555) = 5250.03, *p* < 0.001; CFI = 0.926; RMSEA = 0.046). As shown in Table 2 (Part A) and OSM Figure S2, lower maternal (*d* = −0.324, *p* < 0.001) and paternal affection (*d* = −0.283, *p* = 0.001) at T1 predicted higher T2 constraints, which in turn predicted greater T3 GAD severity: maternal (*d* = 0.463, *p* < 0.001); paternal (*d* = 0.464, *p* < 0.001). Indirect effects via constraints were significant: maternal (*d* = −0.269, *p* = 0.002); paternal (*d* = −0.247, *p* = 0.004), accounting for 69.2% and 51.5% of the total effects, respectively.

**TABLE 2:** T1 childhood parental affection predicting T3 GAD and MDD severity via T2 perceived constraints.

	Maternal affection as predictor		Paternal affection as predictor	
	*β* (95% CI)	*d*	*β* (95% CI)	*d*
A. T3 GAD severity as outcome Regression estimates				
T1 affection to T2 constraints	−0.061*** (−0.092, −0.030)	−0.324	−0.045** (−0.071, −0.018)	−0.283
T2 constraints to T3 GAD severity	0.020*** (0.013, 0.027)	0.463	0.020*** (0.013, 0.027)	0.464
T1 affection to T3 GAD severity	0.003 (−0.001, 0.007)	0.128	0.003 (−0.001, 0.006)	0.139
T1 GAD severity to T3 GAD severity	0.249*** (0.161, 0.338)	0.469	0.249*** (0.160, 0.337)	0.470
Indirect and total effects				
Indirect effect	−0.001** (−0.002, 0.000)	−0.269	−0.001** (−0.002, 0.000)	−0.247
Total effect	0.002 (−0.002, 0.006)	0.076	0.002 (−0.001, 0.005)	0.091
B. T3 MDD severity as outcome				
Regression estimates				
T1 affection to T2 Constraints	−0.061*** (−0.092, −0.030)	−0.396	−0.045** (−0.071, −0.018)	−0.346
T2 constraints to T3 MDD severity	0.031*** (0.021, 0.041)	0.645	0.031*** (0.021, 0.041)	0.647
T1 constraints to T3 MDD severity	−0.003 (−0.009, 0.003)	−0.099	−0.002 (−0.007, 0.004)	−0.067
T1 MDD severity to T3 MDD severity	0.254*** (0.203, 0.305)	1.016	0.255*** (0.204, 0.306)	1.020
Indirect and total effects				
Indirect effect	−0.002** (−0.003, −0.001)	−0.343	−0.001** (−0.002, 0.000)	−0.311
Total effect	−0.005 (−0.011, 0.001)	−0.157	−0.003 (−0.008, 0.002)	−0.120

* *p* < 0.05.

** *p* < 0.01.

*** *p* < 0.001.

Abbreviations: CI, confidence interval; *d*, effect size; GAD, generalized anxiety disorder; MDD, major depressive disorder; *p*, *p*‐value; T1, time 1; T2, time 2; T3, time 3; *β*, factor loading or regression estimate.

### 3.4 T1 Parental Child Affection Predicting T3 MDD Severity via T2 Constraints

The mediation models showed excellent fit for maternal affection (*χ* ^2^(372) = 2471.68, *p* < 0.001; CFI = 0.974; RMSEA = 0.038) and paternal affection (*χ* ^2^(372) = 2107.37, *p* < 0.001; CFI = 0.980; RMSEA = 0.034). As shown in Table 2 (Part B) and OSM Figure S2, lower maternal (*d* = −0.396, *p* < 0.001) and paternal affection (*d* = −0.346, *p* < 0.001) at T1 predicted greater T2 constraints, which in turn predicted higher T3 MDD severity: maternal (*d* = 0.645, *p* < 0.001); paternal (*d* = 0.647, *p* < 0.001). Indirect effects via constraints were significant: maternal (*d* = −0.343, *p* = 0.001); paternal (*d* = −0.311, *p* = 0.003), accounting for 38.0% and 44.9% of the total effects, respectively.

### 3.5 T1 Parental Child Abuse Predicting T3 GAD Severity via T2 Mastery

OSM Figure S3 summarizes the bivariate associations. The mediation models showed acceptable fit for maternal abuse (*χ* ^2^(319) = 4086.87, *p* < 0.001; CFI = 0.902; RMSEA = 0.053) and paternal abuse (*χ* ^2^(319) = 4184.13, *p* < 0.001; CFI = 0.897; RMSEA = 0.054). As shown in Table 3 (Part A), higher maternal abuse (*d* = −0.263, *p* = 0.019), but not paternal abuse (*d* = −0.019, *p* = 0.864), at T1 predicted reduced T2 mastery. Lower mastery then predicted higher T3 GAD severity for both maternal (*d* = −0.265, *p* = 0.018) and paternal abuse paths (*d* = −0.276, *p* = 0.014). Contrary to hypotheses, indirect effects via reduced mastery were not significant for maternal (*d* = 0.187, *p* = 0.094) or paternal abuse (*d* = 0.019, *p* = 0.865).

**TABLE 3:** T1 childhood parental abuse predicting T3 GAD and MDD severity via T2 personal mastery.

	Maternal abuse as predictor		Paternal abuse as predictor	
	*β* (95% CI)	*d*	*β* (95% CI)	*d*
A. T3 GAD severity as outcome Regression estimates				
T1 abuse to T2 mastery	−0.069* (−0.127, −0.011)	−0.263	−0.005 (−0.060, 0.051)	−0.019
T2 mastery to T3 GAD severity	−0.005* (−0.009, −0.001)	−0.265	−0.005* (−0.009, −0.001)	−0.276
T1 abuse to T3 GAD severity	0.004 (−0.001, 0.009)	0.197	0.004 (−0.001, 0.010)	0.172
T1 GAD severity to T3 GAD severity	0.266*** (0.174, 0.358)	0.635	0.265*** (0.174, 0.357)	0.635
Indirect and total effects				
Indirect effect	0.000 (0.000, 0.001)	0.187	0.000 (0.000, 0.000)	0.019
Total effect	0.005 (0.000, 0.010)	0.212	0.004 (−0.001, 0.010)	0.173
B. T3 MDD severity as outcome				
Regression estimates				
T1 abuse to T2 Mastery	−0.069* (−0.127, −0.011)	−0.346	−0.004 (−0.059, 0.052)	−0.019
T2 mastery to T3 MDD severity	−0.007 (−0.015, 0.001)	−0.264	−0.008* (−0.016, 0.000)	−0.299
T1 abuse to T3 MDD severity	0.026*** (0.016, 0.037)	0.741	0.020*** (0.010, 0.030)	0.562
T1 MDD severity to T3 MDD severity	0.260*** (0.208, 0.312)	1.454	0.266*** (0.214, 0.317)	1.494
Indirect and total effects				
Indirect effect	0.000 (0.000, 0.001)	0.205	0.000 (0.000, 0.000)	0.019
Total effect	0.027*** (0.017, 0.037)	0.755	0.020*** (0.010, 0.030)	0.561

* *p* < 0.05.

** *p* < 0.01.

*** *p* < 0.001.

Abbreviations: CI, confidence interval; *d*, effect size; GAD, generalized anxiety disorder; MDD, major depressive disorder; *p*, *p*‐value; T1, time 1; T2, time 2; T3, time 3; *β*, factor loading or regression estimate.

### 3.6 T1 Parental Child Abuse Predicting T3 MDD Severity via T2 Mastery

The mediation models showed excellent fit for maternal abuse (*χ* ^2^(184) = 1111.10, *p* < 0.001; CFI = 0.987; RMSEA = 0.033) and paternal abuse (*χ* ^2^(184) = 1101.80, *p* < 0.001; CFI = 0.987; RMSEA = 0.033). As shown in Table 3 (Part B) and OSM Figure S3, higher maternal abuse (*d* = −0.346, *p* = 0.019), but not paternal abuse (*d* = −0.019, *p* = 0.899), at T1 predicted reduced T2 mastery. Lower T2 mastery predicted higher T3 MDD severity for the paternal abuse path (*d* = −0.299, *p* = 0.043) but not the maternal path (*d* = −0.264, *p* = 0.073). Indirect effects via reduced mastery were not significant for maternal abuse (*d* = 0.205, *p* = 0.164) or paternal abuse (*d* = 0.019, *p* = 0.899).

### 3.7 T1 Parental Child Abuse Predicting T3 GAD Severity via T2 Constraints

The mediation models showed acceptable fit for maternal abuse (*χ* ^2^(429) = 4214.00, *p* < 0.001; CFI = 0.907; RMSEA = 0.046) and paternal abuse (*χ* ^2^(429) = 4308.26, *p* = 0.001; CFI = 0.903; RMSEA = 0.047). As shown in Table 4 (Part A) and OSM Figure S3, higher maternal (*d* = 0.411, *p* < 0.001) and paternal abuse (*d* = 0.286, *p* = 0.003) at T1 predicted greater T2 constraints, which in turn predicted higher T3 GAD severity: maternal (*d* = 0.520, *p* < 0.001); paternal (*d* = 0.526, *p* < 0.001). Indirect effects via constraints were significant for maternal (*d* = 0.319, *p* = 0.001) and paternal abuse (*d* = 0.243, *p* = 0.012), accounting for 39.4% and 30.0% of the total effects, respectively.

**TABLE 4:** T1 childhood parental abuse predicting T3 GAD and MDD severity via T2 perceived constraints.

	Maternal abuse as predictor		Paternal abuse as predictor	
	*β* (95% CI)	*d*	*β* (95% CI)	*d*
A. T3 GAD severity as outcome Regression estimates				
T1 abuse to T2 constraints	0.107*** (0.058, 0.156)	0.411	0.076** (0.026, 0.127)	0.286
T2 constraints to T3 GAD severity	0.020*** (0.012, 0.027)	0.520	0.020*** (0.013, 0.027)	0.526
T1 abuse to T3 GAD severity	0.003 (−0.002, 0.008)	0.125	0.004 (−0.002, 0.009)	0.123
T1 GAD severity to T3 GAD severity	0.244*** (0.156, 0.332)	0.527	0.243*** (0.156, 0.331)	0.526
Indirect and total effects				
Indirect effect	0.002** (0.001, 0.003)	0.319	0.002* (0.000, 0.003)	0.243
Total effect	0.005* (0.000, 0.010)	0.202	0.005 (−0.001, 0.011)	0.171
B. T3 MDD severity as outcome				
Regression estimates				
T1 abuse to T2 constraints	0.105*** (0.056, 0.155)	0.510	0.073** (0.022, 0.123)	0.344
T2 constraints to T3 MDD severity	0.030*** (0.020, 0.039)	0.727	0.031*** (0.021, 0.040)	0.754
T1 abuse to T3 MDD severity	0.024*** (0.014, 0.035)	0.562	0.018*** (0.008, 0.028)	0.431
T1 MDD severity to T3 MDD severity	0.244*** (0.193, 0.295)	1.142	0.249*** (0.198, 0.300)	1.174
Indirect and total effects				
Indirect effect	0.003** (0.001, 0.005)	0.422	0.002* (0.001, 0.004)	0.309
Total effect	0.027*** (0.017, 0.038)	0.634	0.020*** (0.010, 0.031)	0.474

* *p* < 0.05.

** *p* < 0.01.

*** *p* < 0.001.

Abbreviations: CI, confidence interval; *d*, effect size; GAD, generalized anxiety disorder; MDD, major depressive disorder; *p*, *p*‐value; T1, time 1; T2, time 2; T3, time 3; *β*, factor loading or regression estimate.

### 3.8 T1 Parental Child Abuse Predicting T3 MDD Severity via T2 Constraints

The mediation models showed excellent fit for maternal abuse (*χ* ^2^(270) = 1164.28, *p* < 0.001; CFI = 0.988; RMSEA = 0.026) and paternal abuse (*χ* ^2^(270) = 1150.65, *p* < 0.001; CFI = 0.988; RMSEA = 0.026). As shown in Table 4 (Part B) and OSM Figure S3, higher maternal (*d* = 0.510, *p* < 0.001) and paternal abuse (*d* = 0.344, *p* = 0.005) predicted increased constraints, which in turn predicted higher MDD severity: maternal path (*d* = 0.727, *p* < 0.001); paternal path (*d* = 0.754, *p* < 0.001). Indirect effects via constraints were significant for maternal (*d* = 0.422, *p* = 0.001) and paternal abuse (*d* = 0.309, *p* = 0.011), accounting for 11.4% and 11.0% of the total effects, respectively.

### 3.9 Sensitivity Analyses

Tables S2, S3, S4, S5, S6 and S7 reported models adjusting for age, sex, education and race. Indirect effect patterns were identical to the primary analyses, indicating robustness to these covariates.

## 4 Discussion

We tested whether control beliefs (personal mastery and perceived constraints) mediated links between childhood experiences (parental affection and abuse) and adulthood GAD and MDD severity. Findings partially supported our hypotheses: Perceived constraints, not personal mastery, mediated associations between parental experiences and 18‐year GAD and MDD severity. Lower parental affection and higher abuse predicted greater constraints, which in turn predicted elevated GAD and MDD. This extends prior work implicating positive reappraisal coping and self‐esteem as mechanisms linking adverse childhood experiences to adult psychopathology (Ng et al. 2024; Sarkar et al. 2024).

The indirect link between early parental affection (T1) and adulthood GAD and MDD severity (T3) via perceived constraints (T2) aligns with parental emotion socialization theories (Soenens and Vansteenkiste 2020; Watkins et al. 2007). Parental affection and emotional support foster competence (Gurdal et al. 2016; Soenens and Vansteenkiste 2020), promote adaptive emotion regulation (ER) and lower internalizing risk (Ren et al. 2023). Fewer perceived constraints strengthen coping and problem‐solving (Kaiser et al. 2021). These findings parallel evidence that positive emotion socialization enhance effortful control by reframing challenges and reinforcing control (Eisenberg et al. 2001; Valiente et al. 2006). Together, results suggest that early parental affection fosters resilience by shaping adaptive control beliefs that lessen perceived external constraints.

Learned helplessness may explain the observed link between parental abuse (T1) and heightened perceived constraints (T2), contributing to greater adulthood GAD and MDD severity (T3). Adverse childhood experiences can engender self‐defeating coping, undermine adaptive control beliefs and foster helplessness and uncontrollability (Lund et al. 2020; Pilkington et al. 2021). Consequently, individuals may adopt avoidant or sedentary behaviours that worsen long‐term mental health (Zainal et al. 2024). These findings extend prior work linking maladaptive control beliefs, adverse childhood experiences and chronic psychological distress (Bolger and Patterson 2001; Shaw and Krause 2002). Overall, results underscore the enduring effects of parental abuse and the need to target maladaptive control beliefs, such as perceived constraints, to mitigate GAD and MDD risk.

Contrary to expectations, personal mastery did not reliably mediate links between childhood experiences and later GAD or MDD. This aligns with evidence that perceived external barriers exert stronger effects on psychological and physical health than beliefs in personal agency (Infurna et al. 2018; Lachman and Weaver 1998). One account is that chronic stress and helplessness from prolonged maltreatment disrupt executive functions essential for goal‐directed behaviour (Gould et al. 2012; Letkiewicz et al. 2021; Zainal et al. 2025). Alternatively, heightened constraints may erode agency, dampen motivation and impede goal‐setting and pursuit, thereby lowering self‐efficacy (Bandura and Ramachaudran 1994; Infurna and Mayer 2015). Future longitudinal work should test these interpretations and clarify how control beliefs shape mental health outcomes.

The relationship between childhood experiences and adult psychopathology was comparable across maternal and paternal affection or abuse. Significant indirect effects emerged for both parents through perceived constraints but not through personal mastery, with no parent‐specific effects. However, mediation tended to be stronger for maternal predictors (11.4%–69.2%) than paternal ones (11.0%–51.5%). This aligns with evidence that maternal parenting more strongly predicts children's emotional and behavioural difficulties (Meunier et al. 2012), likely due to more frequent maternal interactions (Sanghvi et al. 2023). Supporting this, maternal warmth better predicts reduced child depression over time (del Barrio et al. 2016), and maternal, but not paternal, abuse has been linked to higher psychopathology risk (VanMeter et al. 2021). Further research should clarify the mechanisms underlying the stronger and more enduring influence of maternal experiences.

### 4.1 Clinical Implications

These findings inform targeted interventions for individuals exposed to low parental affection or frequent childhood abuse. Programmes that reduce perceived constraints may mitigate long‐term effects of negative parental experiences, consistent with evidence that strengthening adaptive control beliefs aids trauma recovery (Frazier et al. 2004; Zeligman et al. 2019). The results also underscore the protective role of parental affection (emotional support and safety) in shaping self‐evaluations and well‐being; children who receive warmth and autonomy support show better self‐regulatory competence (Wang and Gai 2024). Incorporating emotion‐socialization strategies into parenting interventions may cultivate positive control beliefs in youth and buffer environmental vulnerabilities linked to adult anxiety and depression. Finally, rather than rigidly applying manuals, target the control facets most relevant to each client to better align treatment with their needs and improve outcomes (Stumpp and Sauer‐Zavala 2022). Longitudinal evidence shows that strengthening mastery leads to stable 2‐year reductions in depressive symptoms (Ulleberg et al. 2021). Together, these results support nuanced approaches to treatment and prevention.

### 4.2 Limitations and Strengths

Several limitations merit note. First, GAD and MDD severity were assessed by trained nonclinicians using DSM‐III‐R criteria (American Psychiatric Association 1987). Because DSM‐III‐R emphasizes somatic features, the mediating role of control facets may depend on symptom operationalization; DSM‐5‐TR foregrounds cognitive and emotional features (e.g., excessive, uncontrollable worry), making these facets especially relevant, so replication with measures consistent with DSM‐5‐TR is warranted (American Psychiatric Association 2022). Second, broader cultural diversity is needed for global generalizability. Third, we focused on perceived control as the mediator given its transdiagnostic relevance (Barlow et al. 2020), but future work should examine it alongside disorder‐specific vulnerabilities such as emotion regulation and stress reactivity (Robinson and Lachman 2017; Weems and Silverman 2006). Fourth, because perceived control was measured in adulthood, it may be influenced by recent stressors. Fifth, childhood abuse and affection were retrospectively reported and thus subject to recall bias, although retrospective reports show good psychometric reliability (Schauss et al. 2021; Zanotti et al. 2018).

This study has several strengths. Its longitudinal design and use of structural equation modelling enhance temporal and causal inference by addressing measurement error (Cole and Maxwell 2003). The large sample provided strong statistical power to test mediation hypotheses. A key contribution lies in examining perceived control through its two orthogonal facets (mastery and constraints; Infurna and Mayer 2015), revealing a more consistent pattern of mediation by constraints. This distinction suggests that early parental behaviours more directly shape perceptions of external limitations, whereas broader social factors may influence self‐efficacy. Finally, by separately analysing parental affection and abuse, the study offers a nuanced understanding of their distinct effects on perceived control and subsequent GAD and MDD severity.

### 4.3 Conclusion

In sum, this 18‐year prospective study identifies perceived constraints as the key mechanism linking low parental affection and high childhood abuse to greater adulthood GAD and MDD severity. Perceived external limitations, rather than personal mastery, confer long‐term risk following adverse or neglectful parenting. Prevention and treatment should prioritize reframing constraining control beliefs and strengthening agency to mitigate long‐term repercussions of early adverse parental experiences.

## Funding

The present manuscript was funded by the National University of Singapore (NUS) Presidential Young Professorship (PYP), Start‐Up Grant and White Space Fund, both of which were awarded to Professor Zainal. Since 1995, the Midlife Development in the United States (MIDUS) study has been funded by the following sources: the John D. and Catherine T. MacArthur Foundation Research Network and the National Institute on Aging (P01‐AG020166; U19‐AG051426). All funding agencies are not responsible for the analyses or interpretations presented here.

## Conflicts of Interest

The authors declare no conflicts of interest.

## Supporting information


**Figure S1:** Longitudinal SEM mediation of T1 parental affection predicting T3 GAD severity via T2 personal mastery, controlling for T1 GAD.
**Figure S2:** Mediation pathways linking T1 parental affection to T3 GAD and MDD severity via T2 personal mastery and perceived constraints.
**Figure S3:** Mediation pathways linking T1 parental abuse to T3 GAD and MDD severity via T2 personal mastery and perceived constraints.
**Table S1:** Sociodemographic details at baseline.
**Table S2:** T1 childhood parental affection predicting T3 GAD and MDD severity via T2 personal mastery.
**Table S3:** T1 childhood parental affection predicting T3 GAD and MDD severity via T2 perceived constraints.
**Table S4:** T1 childhood parental abuse predicting T3 GAD and MDD severity via T2 personal mastery.
**Table S5:** T1 childhood parental abuse predicting T3 GAD and MDD severity via T2 perceived constraints.
**Table S6:** Sensitivity analyses of T1 parental child affection predicting T3 GAD or MDD severity via T2 personal mastery or perceived constraints.
**Table S7:** Sensitivity analyses of T1 parental child abuse predicting T3 GAD or MDD severity via T2 personal mastery or perceived constraints.

## Data Availability

The Midlife Development in the United States (MIDUS) public‐use datasets analysed in this study are available from the Interuniversity Consortium for Political and Social Research (ICPSR) at the University of Michigan. Key study waves include MIDUS 1 (ICPSR02760), MIDUS 2 (ICPSR04652) and MIDUS 3 (ICPSR36346). Access to information, codebooks and documentation can be obtained via the ICPSR website. The R scripts used to clean the data, fit the structural equation models and compute effect sizes are available from the corresponding author upon reasonable request. All primary measures, instruments and study procedures are documented by the MIDUS project and can be retrieved from the ICPSR MIDUS archives, which include detailed questionnaires and technical documentation for each wave.
