# Supplementary material for: When Childhood Control Slips Away: How Parental Affection and Abuse Shape Adult Anxiety and Depression
Source: Clin Psychol Psychother. 2026 Feb 3;33(1):e70232. doi: 10.1002/cpp.70232 (PMC12869133; doi:10.1002/cpp.70232)
Supplement: Supplementary file 1 — Figure S1: Longitudinal SEM mediation of T1 parental affection predicting T3 GAD severity via T2 personal mastery, controlling for T1 GAD. Figure S2: Mediation pathways linking T1 parental affection to T3 GAD and MDD severity via T2 personal mastery and perceived constraints. Figure S3: Mediation pathways linking T1 parental abuse to T3 GAD and MDD severity via T2 personal mastery and perceived constraints. Table S1: Sociodemographic details at baseline. Table S2: T1 childhood parental affection predicting T3 GAD and MDD severity via T2 personal mastery. Table S3: T1 childhood parental affection predicting T3 GAD and MDD severity via T2 perceived constraints. Table S4: T1 childhood parental abuse predicting T3 GAD and MDD severity via T2 personal mastery. Table S5: T1 childhood parental abuse predicting T3 GAD and MDD severity via T2 perceived constraints. Table S6: Sensitivity analyses of T1 parental child affection predicting T3 GAD or MDD severity via T2 personal mastery or perceived constraints. Table S7: Sensitivity analyses of T1 parental child abuse predicting T3 GAD or MDD severity via T2 personal mastery or perceived constraints. [file CPP-33-e70232-s001.docx]

# Online Supplemental Materials (OSM)

**Figure S1**

*Longitudinal SEM Mediation of T1 Parental Affection Predicting T3 GAD Severity via T2 Personal Mastery, controlling for T1 GAD*

**
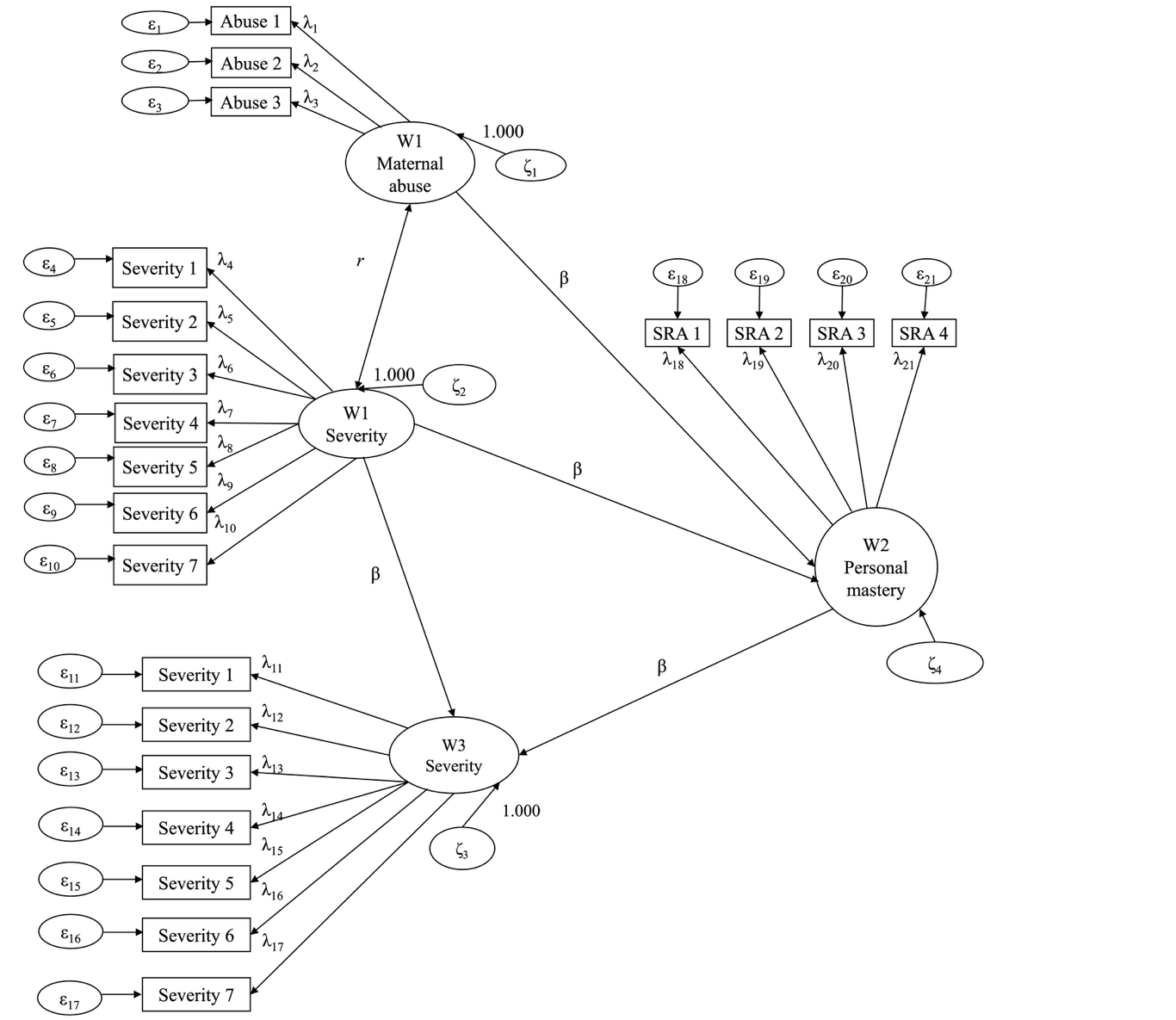
**

*Note.* T1, time 1; T2, time 2; T3, time 3; GAD, generalized anxiety disorder; PM, personal mastery; β = unstandardized beta regression weight with standard error in parentheses; ε = item residual variances; ζ = factor residual variances. The arrows denote the direction of effects, either as part of the measurement model or the structural pathway mediation analysis model.

**Figure S2**

*Mediation Pathways Linking T1 Parental Affection to T3 GAD and MDD Severity via T2 Personal Mastery and Perceived Constraints*


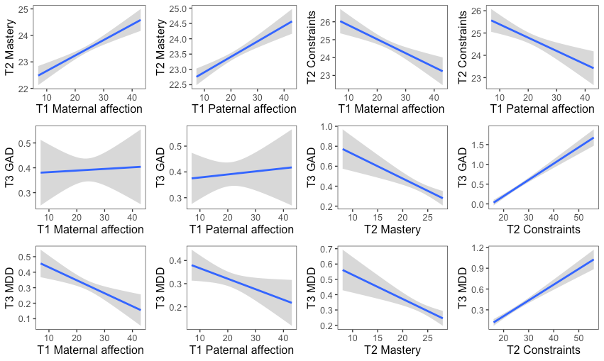


*Note.* T1, time 1; T2, time 2; T3, time 3; GAD, generalized anxiety disorder; MDD, major depressive disorder. Each panel indicates the bivariate association between two variables of interest across all assessment waves. The blue lines denote the linear main effects. The grey ribbons enveloping the blue lines indicate the standard errors.

**Figure S3**

*Mediation Pathways Linking T1 Parental Abuse to T3 GAD and MDD Severity via T2 Personal Mastery and Perceived Constraints*

**
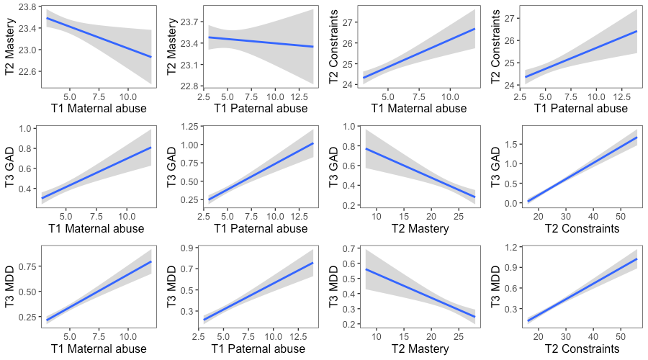
**

*Note.* T1, time 1; T2, time 2; T3, time 3; GAD, generalized anxiety disorder; MDD, major depressive disorder. Each panel indicates the bivariate association between two variables of interest across all assessment waves. The blue lines denote the linear main effects. The grey ribbons enveloping the blue lines indicate the standard errors.

**Table S1**

*Sociodemographic details at baseline*

| Continuous variable | *M* | (*SD*) |
| --- | --- | --- |
| Age (years) | 45.6 | (11.4) |
| Categorical variables | *n* | (%) |
| Gender |  |  |
| Men | 1484 | (45.1) |
| Women | 1810 | (54.9) |
| Education |  |  |
| No school/some grade school | 5 | (0.2) |
| Eighth grade/junior high school | 27 | (0.8) |
| Some high school | 139 | (4.2) |
| General Educational Development (GED) | 31 | (0.9) |
| Graduated from high school | 816 | (24.8) |
| 1 to 2 years of college, no degree yet | 570 | (17.3) |
| 3 or more years of college, no degree yet | 159 | (4.8) |
| Graduated from a 2-year college or vocational school, or associate's degree | 250 | (7.6) |
| Graduated from a 4- or 5-year college, or earned a bachelor's degree | 723 | (21.9) |
| Some graduate school | 113 | (3.4) |
| Master's degree | 313 | (9.5) |
| Ph.D, Ed.D, MD, DDS, LLB, LLD, JD, or other professional degree | 144 | (4.4) |
| Don't know | 4 | (0.1) |
| Race |  |  |
| Respondent does not have self-report data | 129 | (3.9) |
| Multiracial | 28 | (0.8) |
| White | 2956 | (89.7) |
| Black and/or African American | 111 | (3.4) |
| Native American or Aleutian Islander/Eskimo | 11 | (0.3) |
| Asian or Pacific Islander | 16 | (0.5) |
| Other | 43 | (1.3) |

*Note.* Ph.D., Doctor of Philosophy; Ed.D., Doctor of Education; MD, Doctor of Medicine; DDS, Doctor of Dental Surgery; LLB, Bachelor of Laws; LLD, Doctor of Laws; JD, Juris Doctor.

**Table S2**

*T1 Childhood Parental Affection Predicting T3 GAD and MDD Severity via T2 Personal Mastery*

|  | Maternal affection as predictor | |  | Paternal affection as predictor | |
| --- | --- | --- | --- | --- | --- |
|  | β (95% CI) | d |  | β (95% CI) | d |
| **A. T3 GAD severity as outcome**  ***Regression estimates*** |  |  |  |  |  |
| T1 Affection to T2 Mastery | .100^***^ (.065, .134) | 0.550 |  | .091^***^ (.061, .121) | 0.578 |
| T2 Mastery to T3 GAD severity | -.005^*^ (-.010, -.001) | -0.248 |  | -.005^*^ (-.010, -.001) | -0.249 |
| T1 Affection to T3 GAD severity | .002 (-.001, .006) | 0.119 |  | .002 (-.001, .006) | 0.138 |
| T1 GAD severity to T3 GAD severity | .272^***^ (.179, .365) | 0.555 |  | .271^***^ (.179, .364) | 0.555 |
| ***Indirect and total effects*** |  |  |  |  |  |
| Indirect effect | -.001^*^ (-.001, .000) | -0.227 |  | .000^*^ (-.001, .000) | -0.232 |
| Total effect | .002 (-.002, .006) | 0.094 |  | .002 (-.001, .005) | 0.111 |
| **B. T3 MDD severity as outcome** |  |  |  |  |  |
| ***Regression estimates*** |  |  |  |  |  |
| T1 Affection to T2 Mastery | .100^***^ (.066, .134) | 0.694 |  | .091^***^ (.061, .121) | 0.728 |
| T2 Mastery to T3 MDD severity | -.008 (-.015, .000) | -0.227 |  | -.008 (-.016, .000) | -0.230 |
| T1 Affection to T3 MDD severity | -.004 (-.010, .002) | -0.149 |  | -.002 (-.008, .003) | -0.102 |
| T1 MDD severity to T3 MDD severity | .271^***^ (.219, .323) | 1.254 |  | .272^***^ (.221, .324) | 1.259 |
| ***Indirect and total effects*** |  |  |  |  |  |
| Indirect effect | -.001 (-.002, .000) | -0.213 |  | -.001 (-.001, .000) | -0.216 |
| Total effect | -.005 (-.011, .002) | -0.176 |  | -.003 (-.008, .002) | -0.134 |

*Note.* * *p* < .05*; ** p* < .01*; *** p* < .001.

T1, time 1; T2, time 2; T3, time 3; GAD, generalized anxiety disorder; MDD, major depressive disorder; β, factor loading or regression estimate; CI, confidence interval; *p*, p-value; d, effect size.

**Table S3**

*T1 Childhood Parental Affection Predicting T3 GAD and MDD Severity via T2 Perceived Constraints*

|  | Maternal affection as predictor | |  | Paternal affection as predictor | |
| --- | --- | --- | --- | --- | --- |
|  | β (95% CI) | d |  | β (95% CI) | d |
| **A. T3 GAD severity as outcome**  ***Regression estimates*** |  |  |  |  |  |
| T1 Affection to T2 Constraints | -.061^***^ (-.092, -.030) | -0.324 |  | -.045^**^ (-.071, -.018) | -0.283 |
| T2 Constraints to T3 GAD severity | .020^***^ (.013, .027) | 0.463 |  | .020^***^ (.013, .027) | 0.464 |
| T1 Affection to T3 GAD severity | .003 (-.001, .007) | 0.128 |  | .003 (-.001, .006) | 0.139 |
| T1 GAD severity to T3 GAD severity | .249^***^ (.161, .338) | 0.469 |  | .249^***^ (.160, .337) | 0.470 |
| ***Indirect and total effects*** |  |  |  |  |  |
| Indirect effect | -.001^**^ (-.002, .000) | -0.269 |  | -.001^**^ (-.002, .000) | -0.247 |
| Total effect | .002 (-.002, .006) | 0.076 |  | .002 (-.001, .005) | 0.091 |
| **B. T3 MDD severity as outcome** |  |  |  |  |  |
| ***Regression estimates*** |  |  |  |  |  |
| T1 Affection to T2 Constraints | -.061^***^ (-.092, -.030) | -0.396 |  | -.045^**^ (-.071, -.018) | -0.346 |
| T2 Constraints to T3 MDD severity | .031^***^ (.021, .041) | 0.645 |  | .031^***^ (.021, .041) | 0.647 |
| T1 Constraints to T3 MDD severity | -.003 (-.009, .003) | -0.099 |  | -.002 (-.007, .004) | -0.067 |
| T1 MDD severity to T3 MDD severity | .254^***^ (.203, .305) | 1.016 |  | .255^***^ (.204, .306) | 1.020 |
| ***Indirect and total effects*** |  |  |  |  |  |
| Indirect effect | -.002^**^ (-.003, -.001) | -0.343 |  | -.001^**^ (-.002, .000) | -0.311 |
| Total effect | -.005 (-.011, .001) | -0.157 |  | -.003 (-.008, .002) | -0.120 |

*Note.* * *p* < .05*; ** p* < .01*; *** p* < .001.

T1, time 1; T2, time 2; T3, time 3; GAD, generalized anxiety disorder; MDD, major depressive disorder; β, factor loading or regression estimate; CI, confidence interval; *p*, p-value; d, effect size.

**Table S4**

*T1 Childhood Parental Abuse Predicting T3 GAD and MDD Severity via T2 Personal Mastery*

|  | Maternal abuse as predictor | |  | Paternal abuse as predictor | |
| --- | --- | --- | --- | --- | --- |
|  | β (95% CI) | d |  | β (95% CI) | d |
| **A. T3 GAD severity as outcome**  ***Regression estimates*** |  |  |  |  |  |
| T1 Abuse to T2 Mastery | -.069^*^ (-.127, -.011) | -0.263 |  | -.005 (-.060, .051) | -0.019 |
| T2 Mastery to T3 GAD severity | -.005^*^ (-.009, -.001) | -0.265 |  | -.005^*^ (-.009, -.001) | -0.276 |
| T1 Abuse to T3 GAD severity | .004 (-.001, .009) | 0.197 |  | .004 (-.001, .010) | 0.172 |
| T1 GAD severity to T3 GAD severity | .266^***^ (.174, .358) | 0.635 |  | .265^***^ (.174, .357) | 0.635 |
| ***Indirect and total effects*** |  |  |  |  |  |
| Indirect effect | .000 (.000, .001) | 0.187 |  | .000 (.000, .000) | 0.019 |
| Total effect | .005 (.000, .010) | 0.212 |  | .004 (-.001, .010) | 0.173 |
| **B. T3 MDD severity as outcome** |  |  |  |  |  |
| ***Regression estimates*** |  |  |  |  |  |
| T1 Abuse to T2 Mastery | -.069^*^ (-.127, -.011) | -0.346 |  | -.004 (-.059, .052) | -0.019 |
| T2 Mastery to T3 MDD severity | -.007 (-.015, .001) | -0.264 |  | -.008^*^ (-.016, .000) | -0.299 |
| T1 Abuse to T3 MDD severity | .026^***^ (.016, .037) | 0.741 |  | .020^***^ (.010, .030) | 0.562 |
| T1 MDD severity to T3 MDD severity | .260^***^ (.208, .312) | 1.454 |  | .266^***^ (.214, .317) | 1.494 |
| ***Indirect and total effects*** |  |  |  |  |  |
| Indirect effect | .000 (.000, .001) | 0.205 |  | .000 (.000, .000) | 0.019 |
| Total effect | .027^***^ (.017, .037) | 0.755 |  | .020^***^ (.010, .030) | 0.561 |

*Note.* * *p* < .05*; ** p* < .01*; *** p* < .001.

T1, time 1; T2, time 2; T3, time 3; GAD, generalized anxiety disorder; MDD, major depressive disorder; β, factor loading or regression estimate; CI, confidence interval; *p*, p-value; d, effect size.

**Table S5**

*T1 Childhood Parental Abuse Predicting T3 GAD and MDD Severity via T2 Perceived Constraints*

|  | Maternal abuse as predictor | |  | Paternal abuse as predictor | |
| --- | --- | --- | --- | --- | --- |
|  | β (95% CI) | d |  | β (95% CI) | d |
| **A. T3 GAD severity as outcome**  ***Regression estimates*** |  |  |  |  |  |
| T1 Abuse to T2 Constraints | .107^***^ (.058, .156) | 0.411 |  | .076^**^ (.026, .127) | 0.286 |
| T2 Constraints to T3 GAD severity | .020^***^ (.012, .027) | 0.520 |  | .020^***^ (.013, .027) | 0.526 |
| T1 Abuse to T3 GAD severity | .003 (-.002, .008) | 0.125 |  | .004 (-.002, .009) | 0.123 |
| T1 GAD severity to T3 GAD severity | .244^***^ (.156, .332) | 0.527 |  | .243^***^ (.156, .331) | 0.526 |
| ***Indirect and total effects*** |  |  |  |  |  |
| Indirect effect | .002^**^ (.001, .003) | 0.319 |  | .002^*^ (.000, .003) | 0.243 |
| Total effect | .005^*^ (.000, .010) | 0.202 |  | .005 (-.001, .011) | 0.171 |
| **B. T3 MDD severity as outcome** |  |  |  |  |  |
| ***Regression estimates*** |  |  |  |  |  |
| T1 Abuse to T2 Constraints | .105^***^ (.056, .155) | 0.510 |  | .073^**^ (.022, .123) | 0.344 |
| T2 Constraints to T3 MDD severity | .030^***^ (.020, .039) | 0.727 |  | .031^***^ (.021, .040) | 0.754 |
| T1 Abuse to T3 MDD severity | .024^***^ (.014, .035) | 0.562 |  | .018^***^ (.008, .028) | 0.431 |
| T1 MDD severity to T3 MDD severity | .244^***^ (.193, .295) | 1.142 |  | .249^***^ (.198, .300) | 1.174 |
| ***Indirect and total effects*** |  |  |  |  |  |
| Indirect effect | .003^**^ (.001, .005) | 0.422 |  | .002^*^ (.001, .004) | 0.309 |
| Total effect | .027^***^ (.017, .038) | 0.634 |  | .020^***^ (.010, .031) | 0.474 |

*Note.* * *p* < .05*; ** p* < .01*; *** p* < .001.

T1, time 1; T2, time 2; T3, time 3; GAD, generalized anxiety disorder; MDD, major depressive disorder; β, factor loading or regression estimate; CI, confidence interval; *p*, p-value; d, effect size.

**Table S6**

*Sensitivity Analyses of T1 Parental Child Affection Predicting T3 GAD or MDD severity via T2 Personal Mastery or Perceived Constraints*

| **Exposure: T1 Parental child affection** | | | | | | | | | |
| --- | --- | --- | --- | --- | --- | --- | --- | --- | --- |
| Mediator | Outcome | Parent | Model | Indirect effects | |  | Total effects | | Percentage |
|  |  |  |  | Estimate | *p* |  | Estimate | *p* | of Mediation |
| T2 Mastery | T3 GAD severity | Maternal | Base | -0.0008 | .005 |  | 0.0020 | .302 | 37.10 |
| T2 Mastery | T3 GAD severity | Maternal | Age (years) | -0.0008 | .004 |  | 0.0020 | .302 | 38.32 |
| T2 Mastery | T3 GAD severity | Maternal | Sex | -0.0007 | .008 |  | 0.0020 | .301 | 33.14 |
| T2 Mastery | T3 GAD severity | Maternal | Education | -0.0008 | .004 |  | 0.0020 | .301 | 37.44 |
| T2 Mastery | T3 GAD severity | Maternal | Race | -0.0008 | .005 |  | 0.0020 | .302 | 37.26 |
| T2 Mastery | T3 GAD severity | Paternal | Base | -0.0007 | .004 |  | 0.0017 | .277 | 39.19 |
| T2 Mastery | T3 GAD severity | Paternal | Age (years) | -0.0007 | .003 |  | 0.0017 | .280 | 40.61 |
| T2 Mastery | T3 GAD severity | Paternal | Sex | -0.0006 | .006 |  | 0.0017 | .276 | 35.11 |
| T2 Mastery | T3 GAD severity | Paternal | Education | -0.0007 | .003 |  | 0.0017 | .276 | 39.54 |
| T2 Mastery | T3 GAD severity | Paternal | Race | -0.0007 | .004 |  | 0.0017 | .277 | 39.36 |
| T2 Mastery | T3 MDD severity | Maternal | Base | -0.0012 | .014 |  | -0.0048 | .142 | 25.74 |
| T2 Mastery | T3 MDD severity | Maternal | Age (years) | -0.0013 | .012 |  | -0.0048 | .143 | 26.52 |
| T2 Mastery | T3 MDD severity | Maternal | Sex | -0.0011 | .027 |  | -0.0048 | .142 | 22.64 |
| T2 Mastery | T3 MDD severity | Maternal | Education | -0.0013 | .013 |  | -0.0048 | .142 | 25.99 |
| T2 Mastery | T3 MDD severity | Maternal | Race | -0.0012 | .014 |  | -0.0048 | .142 | 25.82 |
| T2 Mastery | T3 MDD severity | Paternal | Base | -0.0011 | .011 |  | -0.0023 | .386 | 49.04 |
| T2 Mastery | T3 MDD severity | Paternal | Age (years) | -0.0012 | .009 |  | -0.0023 | .386 | 50.40 |
| T2 Mastery | T3 MDD severity | Paternal | Sex | -0.0010 | .021 |  | -0.0023 | .385 | 43.37 |
| T2 Mastery | T3 MDD severity | Paternal | Education | -0.0011 | .010 |  | -0.0023 | .386 | 49.50 |
| T2 Mastery | T3 MDD severity | Paternal | Race | -0.0011 | .011 |  | -0.0023 | .386 | 49.17 |
| T2 Constraints | T3 GAD severity | Maternal | Base | -0.0018 | .000 |  | 0.0022 | .262 | 80.42 |
| T2 Constraints | T3 GAD severity | Maternal | Age (years) | -0.0018 | .000 |  | 0.0022 | .262 | 81.08 |
| T2 Constraints | T3 GAD severity | Maternal | Sex | -0.0017 | .000 |  | 0.0022 | .261 | 76.73 |
| T2 Constraints | T3 GAD severity | Maternal | Education | -0.0017 | .000 |  | 0.0022 | .262 | 78.93 |
| T2 Constraints | T3 GAD severity | Maternal | Race | -0.0018 | .000 |  | 0.0022 | .262 | 80.98 |
| T2 Constraints | T3 GAD severity | Paternal | Base | -0.0013 | .001 |  | 0.0018 | .250 | 72.35 |
| T2 Constraints | T3 GAD severity | Paternal | Age (years) | -0.0013 | .001 |  | 0.0018 | .252 | 73.20 |
| T2 Constraints | T3 GAD severity | Paternal | Sex | -0.0013 | .001 |  | 0.0018 | .250 | 69.06 |
| T2 Constraints | T3 GAD severity | Paternal | Education | -0.0013 | .001 |  | 0.0018 | .250 | 70.96 |
| T2 Constraints | T3 GAD severity | Paternal | Race | -0.0013 | .001 |  | 0.0018 | .250 | 72.85 |
| T2 Constraints | T3 MDD severity | Maternal | Base | -0.0028 | .000 |  | -0.0046 | .157 | 61.38 |
| T2 Constraints | T3 MDD severity | Maternal | Age (years) | -0.0029 | .000 |  | -0.0046 | .158 | 61.91 |
| T2 Constraints | T3 MDD severity | Maternal | Sex | -0.0027 | .000 |  | -0.0046 | .157 | 57.76 |
| T2 Constraints | T3 MDD severity | Maternal | Education | -0.0028 | .000 |  | -0.0046 | .157 | 60.32 |
| T2 Constraints | T3 MDD severity | Maternal | Race | -0.0029 | .000 |  | -0.0046 | .157 | 61.68 |
| T2 Constraints | T3 MDD severity | Paternal | Base | -0.0021 | .000 |  | -0.0022 | .405 | 95.69 |
| T2 Constraints | T3 MDD severity | Paternal | Age (years) | -0.0021 | .000 |  | -0.0022 | .406 | 96.46 |
| T2 Constraints | T3 MDD severity | Paternal | Sex | -0.0020 | .001 |  | -0.0022 | .405 | 90.10 |
| T2 Constraints | T3 MDD severity | Paternal | Education | -0.0021 | .000 |  | -0.0022 | .405 | 94.02 |
| T2 Constraints | T3 MDD severity | Paternal | Race | -0.0021 | .000 |  | -0.0022 | .405 | 96.15 |

*Note.* T1, time 1; T2, time 2; T3, time 3; GAD, generalized anxiety disorder; MDD, major depressive disorder.

**Table S7**

*Sensitivity Analyses of T1 Parental Child Abuse Predicting T3 GAD or MDD severity via T2 Personal Mastery or Perceived Constraints*

| **Exposure: T1 Parental child abuse** | | | | | | | | | |
| --- | --- | --- | --- | --- | --- | --- | --- | --- | --- |
| Mediator | Outcome | Parent | Model | Indirect effects | |  | Total effects | | Percentage |
|  |  |  |  | Estimate | *p* |  | Estimate | *p* | of Mediation |
| T2 Mastery | T3 GAD severity | Maternal | Base | 0.0007 | .037 |  | 0.0046 | .044 | 15.14 |
| T2 Mastery | T3 GAD severity | Maternal | Age (years) | 0.0007 | .035 |  | 0.0046 | .044 | 15.15 |
| T2 Mastery | T3 GAD severity | Maternal | Sex | 0.0006 | .040 |  | 0.0046 | .044 | 14.08 |
| T2 Mastery | T3 GAD severity | Maternal | Education | 0.0007 | .036 |  | 0.0046 | .044 | 15.15 |
| T2 Mastery | T3 GAD severity | Maternal | Race | 0.0007 | .036 |  | 0.0046 | .044 | 15.21 |
| T2 Mastery | T3 GAD severity | Paternal | Base | 0.0003 | .188 |  | 0.0039 | .130 | 7.31 |
| T2 Mastery | T3 GAD severity | Paternal | Age (years) | 0.0003 | .185 |  | 0.0039 | .129 | 7.27 |
| T2 Mastery | T3 GAD severity | Paternal | Sex | 0.0003 | .195 |  | 0.0039 | .130 | 6.73 |
| T2 Mastery | T3 GAD severity | Paternal | Education | 0.0003 | .187 |  | 0.0039 | .129 | 7.30 |
| T2 Mastery | T3 GAD severity | Paternal | Race | 0.0003 | .187 |  | 0.0039 | .130 | 7.36 |
| T2 Mastery | T3 MDD severity | Maternal | Base | 0.0012 | .067 |  | 0.0251 | .000 | 4.61 |
| T2 Mastery | T3 MDD severity | Maternal | Age (years) | 0.0012 | .065 |  | 0.0251 | .000 | 4.63 |
| T2 Mastery | T3 MDD severity | Maternal | Sex | 0.0011 | .082 |  | 0.0251 | .000 | 4.20 |
| T2 Mastery | T3 MDD severity | Maternal | Education | 0.0012 | .066 |  | 0.0251 | .000 | 4.61 |
| T2 Mastery | T3 MDD severity | Maternal | Race | 0.0012 | .066 |  | 0.0251 | .000 | 4.64 |
| T2 Mastery | T3 MDD severity | Paternal | Base | 0.0004 | .256 |  | 0.0170 | .000 | 2.49 |
| T2 Mastery | T3 MDD severity | Paternal | Age (years) | 0.0004 | .254 |  | 0.0170 | .000 | 2.48 |
| T2 Mastery | T3 MDD severity | Paternal | Sex | 0.0004 | .268 |  | 0.0170 | .000 | 2.27 |
| T2 Mastery | T3 MDD severity | Paternal | Education | 0.0004 | .256 |  | 0.0170 | .000 | 2.49 |
| T2 Mastery | T3 MDD severity | Paternal | Race | 0.0004 | .255 |  | 0.0170 | .000 | 2.51 |
| T2 Constraints | T3 GAD severity | Maternal | Base | 0.0042 | .037 |  | 0.0117 | .078 | 35.52 |
| T2 Constraints | T3 GAD severity | Maternal | Age (years) | 0.0042 | .036 |  | 0.0117 | .078 | 36.03 |
| T2 Constraints | T3 GAD severity | Maternal | Sex | 0.0038 | .039 |  | 0.0117 | .079 | 32.32 |
| T2 Constraints | T3 GAD severity | Maternal | Education | 0.0041 | .038 |  | 0.0118 | .077 | 34.49 |
| T2 Constraints | T3 GAD severity | Maternal | Race | 0.0041 | .036 |  | 0.0117 | .079 | 35.36 |
| T2 Constraints | T3 GAD severity | Paternal | Base | 0.0032 | .075 |  | 0.0043 | .481 | 75.58 |
| T2 Constraints | T3 GAD severity | Paternal | Age (years) | 0.0033 | .075 |  | 0.0043 | .478 | 76.19 |
| T2 Constraints | T3 GAD severity | Paternal | Sex | 0.0030 | .077 |  | 0.0043 | .482 | 71.26 |
| T2 Constraints | T3 GAD severity | Paternal | Education | 0.0032 | .075 |  | 0.0044 | .471 | 72.68 |
| T2 Constraints | T3 GAD severity | Paternal | Race | 0.0032 | .074 |  | 0.0043 | .482 | 75.26 |
| T2 Constraints | T3 MDD severity | Maternal | Base | -0.0012 | .036 |  | -0.0047 | .238 | 25.56 |
| T2 Constraints | T3 MDD severity | Maternal | Age (years) | -0.0012 | .034 |  | -0.0047 | .239 | 25.80 |
| T2 Constraints | T3 MDD severity | Maternal | Sex | -0.0011 | .049 |  | -0.0047 | .238 | 23.27 |
| T2 Constraints | T3 MDD severity | Maternal | Education | -0.0012 | .035 |  | -0.0047 | .238 | 25.58 |
| T2 Constraints | T3 MDD severity | Maternal | Race | -0.0012 | .036 |  | -0.0047 | .238 | 25.50 |
| T2 Constraints | T3 MDD severity | Paternal | Base | -0.0009 | .065 |  | -0.0026 | .412 | 33.44 |
| T2 Constraints | T3 MDD severity | Paternal | Age (years) | -0.0009 | .065 |  | -0.0026 | .412 | 33.22 |
| T2 Constraints | T3 MDD severity | Paternal | Sex | -0.0008 | .090 |  | -0.0026 | .412 | 29.98 |
| T2 Constraints | T3 MDD severity | Paternal | Education | -0.0009 | .064 |  | -0.0026 | .412 | 33.48 |
| T2 Constraints | T3 MDD severity | Paternal | Race | -0.0009 | .065 |  | -0.0026 | .412 | 33.39 |

*Note.* T1, time 1; T2, time 2; T3, time 3; GAD, generalized anxiety disorder; MDD, major depressive disorder.
